# The Effectiveness of Periodontally Accelerated Osteogenic Orthodontics (PAOO) in Accelerating Tooth Movement and Supporting Alveolar Bone Thickness During Orthodontic Treatment: A Systematic Review

**DOI:** 10.7759/cureus.24985

**Published:** 2022-05-14

**Authors:** Hallaj I Alsino, Mohammad Y Hajeer, Ahmad S Burhan, Issam Alkhouri, Khaldoun Darwich

**Affiliations:** 1 Department of Orthodontics, University of Damascus Faculty of Dentistry, Damascus, SYR; 2 Department of Oral and Maxillofacial Surgery, University of Damascus Faculty of Dentistry, Damascus, SYR

**Keywords:** patient-reported outcome measures, acceleration of leveling and alignment, accelerated tooth movement, bone density, alveolar bone thickness, bone grafts, conventional orthodontic treatment, corticotomy, periodontally accelerated osteogenic orthodontics (paoo)

## Abstract

The current review aimed to critically and systematically evaluate the available evidence regarding the effectiveness of periodontally accelerated osteogenic orthodontics (PAOO) in accelerating orthodontic tooth movement and supporting the alveolar bone. Additionally, this review aimed to analyze the untoward effects of this procedure and the patient-reported outcome measures.

A comprehensive electronic search was performed on 10 databases in addition to a manual search to retrieve all relevant studies. Randomized controlled trials (RCTs) were only included in this review. The interventional group was the PAOO procedure, whereas the control group was either a non-accelerated traditional fixed orthodontic treatment or an accelerated treatment using any other intervention. The Cochrane risk of bias tool for randomized controlled trials (RoB 2) was employed to estimate the risk of bias in the included studies.

The current review included eight RCTs evaluating 175 participants (63 males and 112 females) with a mean age ranging from 18.8 to 29.6 years. Five of them assessed the effectiveness of PAOO versus traditional orthodontic treatment, i.e. without any adjuvant surgical intervention. At the same time, the remaining three studies evaluated the effectiveness of PAOO versus corticotomy-only as an adjunctive procedure. The PAOO accelerated the leveling and alignment stage from 39% to 47% and accelerated the retraction of the upper anterior teeth from 41% to 61% compared to conventional orthodontic treatment. One study only indicated that PAOO reduced treatment time by 30.3% versus a corticotomy-only as an adjunctive procedure. No significant side effects have been reported with the PAOO procedure.

The PAOO procedure was effective in accelerating orthodontic movement and tended to increase the thickness of the alveolar bone. But most periodontal outcome measures regarding PAOO application were not comprehensively covered in the included trials.

## Introduction and background

Treatment of moderate to severe cases of malocclusion requires more than a year and a half using fixed orthodontics [1]. Because of the long treatment period, many orthodontic patients may refuse treatment [2]. Reducing the duration of orthodontic treatment has recently become a goal of orthodontists by increasing the rate of movement of the tooth because of the common side effects that accompany lengthy orthodontic treatments such as root resorption, gingival inflammation, decalcification, and dental caries [3,4].

Different methods have been used to reduce the duration of orthodontic treatment. Still, surgical procedures may appear to be more clinical use, with results that may seem better in reducing orthodontic treatment time [5]. A surgical procedure that involved both osteotomy and corticotomy was first described by Köle in 1959. Köle claimed the movement of the "blocks of bone" is the cause of the accelerated tooth movement by selective corticotomy [6].

However, recent evidence suggests the increase in the rate of tooth movement can be attributed to the localized osteoporosis state as a part of a healing event called the regional acceleratory phenomenon (RAP) [7]. Several surgical procedures have been used to accelerate tooth movement by taking advantage of the RAP phenomenon, such as conventional corticotomy [8,9], piezocision-based flapless corticotomy [10,11], corticision [12], and laser-assisted flapless corticotomy [13].

Wilcko et al. emphasized the RAP concept through radiological evidence of the state of osteoporosis in the alveolar bone treated with corticotomy. This was supported by data from computerized tomography (CT) that rejected the concept of "blocks of bone" movement [14]. Periodontally accelerated osteogenic orthodontics (PAOO) has gained popularity and acceptance because its results may be safe and effective in addition to the benefits versus conventional orthodontic treatment [14]. He claimed that PAOO has better results than traditional orthodontic treatment because it is suitable for increasing tooth movement without increasing the risk of absorbing the apical root and increasing the alveolar bone, and reshaping it simultaneously. Many PAOO clinical case reports claim it is a suitable treatment procedure for patients who wish to end orthodontic treatment in a short period and reduce the risk of root absorption and an increase in the thickness of the alveolar bone [14-16]. However, only a few clinical trials on PAOO have been released [17-19].

To the best of our knowledge, there is only one systematic review on PAOO's effectiveness in improving periodontal outcomes and reducing treatment duration [20], which discussed the course of orthodontic treatment, root length, bone density, and pocket depth without evaluating skeletal and soft tissue variables. Also, this systematic review did consider the possible positive outcomes of PAOO on preventing the formation of alveolar bone defects such as 'dehiscence' and 'fenestration'. The electronic database search was confined to four databases with only five retrieved studies conducted in 2017, i.e., four years have passed. Consequently, this review aims to critically and systematically appraise the available evidence regarding the effectiveness of PAOO in inducing rapid orthodontic tooth movement and probably improving the structure of the alveolar bone with an estimation of the untoward effects of this procedure.

## Review

Preliminary search before review commencement

Before writing the final systematic review protocol, a preliminary PubMed search was performed to verify similar systematic reviews and explore articles relevant to the review topic. The protocol was recorded during the early stages of this review in PROSPERO (CRD42021274477). Using the Preferred Reporting Items for Systematic Reviews and Meta-Analyses (PRISMA) [21], as well as the checklist and the Cochrane Handbook for Systematic reviews of interventions version 5.1.0 [22], this systematic review was written up and submitted.

Eligibility criteria

The PICOS framework was set as follows: Participants: Healthy male and female patients over 14 years of age with any type of malocclusion and any ethnic group who received treatment with fixed orthodontic appliances. Intervention: Fixed orthodontic treatment associated with periodontally accelerated osteogenic orthodontics (PAOO) as an adjunctive procedure. Comparison: Fixed orthodontic treatment without any surgical interventions. If the treatment was associated with any adjunctive surgical procedure, this procedure should not be a PAOO procedure. Study design: Randomized controlled trials (RCTs) published between January 1998 and August 2021, in the English language only. Outcome measures: Treatment duration and alveolar bone thickness were the primary outcomes, whereas the secondary outcome measures included bone defects, loss of attachment, probing depth, root resorption, and stability of treatment in the long term.

The excluded studies included the following: Case reports or case series reports, retrospective studies, in-vitro studies, animal studies, non-English language trials, editorials articles, personal opinions, articles describing the therapeutic technique, studies that do not clearly describe the included sample, split-mouth-design studies, studies using lingual or self-ligating brackets in comparison with labial or conventional-ligating brackets, and an age range greater than ten years between the youngest and the oldest patient in any studied group.

Search strategy

The electronic search was performed within the literature published on August 19, 2021, using PubMed®, Medline®, Google™ Scholar, the Cochrane Central Register of Controlled Trials (CENTRAL), EBSCO eBooks™, OVID® SP, Web of Science™, Embase®, Scopus®, and OpenGrey. The databases were searched for papers published between January 1998 and August 2021. A manual search was performed within the time frame specified in the Angle Orthodontist, the American Journal of Orthodontics and Dentofacial Orthopedics, the European Journal of Orthodontics, the Journal of Orthodontics and Craniofacial Research, and the Journal of Orthodontics. The search included ClinicalTrials.gov and the World Health Organization International Clinical Trials Registry Platform Search Portal (ICTRP) for all clinical trials that were completed, in progress, or not yet published. The search strategy within the databases and journals is presented in Table 1.

**Table 1:** Electronic search strategy within databases PAOO: periodontally accelerated osteogenic orthodontics

No.	Database	Search strategy	Results
1	CENTRAL (The Cochrane Library)	#1 "Periodontally Accelerated Osteogenic Orthodontics" OR "Accelerated Osteogenic Orthodontics" OR "Corticotomy-Assisted Orthodontic Treatment" OR "Corticotomy" OR "Regional Acceleratory Phenomenon" OR "piezoelectric surgery" OR "alveolar augmentation" #2 "orthodontic treatment" OR "orthodontic therapy" OR "crowded teeth" OR "extraction teeth" OR "non-extraction teeth" #3 #1 AND #2	64
2	PubMed	#1 "Periodontally Accelerated Osteogenic Orthodontics*" OR "Accelerated Osteogenic Orthodontics" OR "Wilckodontics" OR "Rapid orthodontics" OR "surgically assisted orthodontics" OR "Corticotomy-assisted orthodontic treatment" OR "tooth movement acceleration" OR "Corticotomy" OR "Selective alveolar decortications" OR "Surgical facilitated orthodontics" OR "Periodontal decortication" OR "Regional Acceleratory Phenomenon" OR "Alveolar corticotomies" OR "Piezoelectric surgery" OR "alveolar augmentation" #2 "Orthodontic treatment*" OR "orthodontic therapy" OR "Skeletal class I" OR "Skeletal class II" OR "Skeletal class III" OR "Crowded teeth" OR "extraction teeth" OR "non-extraction teeth" #3 "Bone graft*" OR "Autograft" OR "Allograft" OR "Xenograft" OR "Synthetic Bone Substitutes" OR "Composite grafts" #4 #1 AND #2 AND #3	84
3	Scopus	#1 TITLE-ABS-KEY (orthodontic* OR "orthodontic treatment" OR "orthodontic therapy" OR "crowded teeth" OR "extraction teeth" OR "non-extraction teeth") #2 TITLE-ABS-KEY ("Periodontally Accelerated Osteogenic Orthodontics" OR "Accelerated Osteogenic Orthodontics" OR "Corticotomy-Assisted Orthodontic Treatment" OR "Corticotomy" OR "Regional Acceleratory Phenomenon" OR "piezoelectric surgery" OR "alveolar augmentation") #3 #1 AND #2	77
4	OVID® SP	#1 Periodontally Accelerated Osteogenic Orthodontics* OR Accelerated Osteogenic Orthodontics* OR Regional Acceleratory Phenomenon* OR Corticotomy-Assisted Orthodontic Treatment* #2 Orthodontic treatment* or orthodontic therapy* #3 Bone graft* #4 #1 AND #2 AND #3	82
5	Google Scholar	#1 ("Periodontally Accelerated Osteogenic Orthodontics" OR "Accelerated Osteogenic Orthodontics" OR "Wilckodontics" OR "Rapid orthodontics" OR "surgically assisted orthodontics" OR "Corticotomy-assisted orthodontic treatment" OR "tooth movement acceleration")	43
6	World Health Organization (WHO) International Clinical Trials Registry Platform (ICTRP)	"Periodontally Accelerated Osteogenic Orthodontics" OR "PAOO"	8
7	ClinicalTrials.gov	"Periodontally Accelerated Osteogenic Orthodontics" OR "PAOO"	5
8	European Journal of Orthodontics	#1 Periodontally Accelerated Osteogenic Orthodontics OR Accelerated Osteogenic Orthodontics OR Wilckodontics OR Regional Acceleratory Phenomenon #2 Orthodontic treatment OR orthodontic therapy #3 Bone graft #4 #1 AND #2 AND #3	7
9	Orthodontics and Craniofacial Research	"Periodontally Accelerated Osteogenic Orthodontics" OR "PAOO"	8
10	American Journal of Orthodontics and Dentofacial Orthopedics	#1 Periodontally Accelerated Osteogenic Orthodontics OR Accelerated Osteogenic Orthodontics OR Wilckodontics OR Regional Acceleratory Phenomenon #2 Orthodontic treatment OR orthodontic therapy #3 Bone graft #4 #1 AND #2 AND #3	23
11	Journal of Orthodontics	Periodontally Accelerated Osteogenic Orthodontics OR PAOO	2
12	the Angle Orthodontist	#1 Periodontally Accelerated Osteogenic Orthodontics OR Accelerated Osteogenic Orthodontics OR Wilckodontics #2 Orthodontic treatment #3 Bone graft #4 #1 AND #2 AND #3	11

Study selection

All articles were evaluated by two reviewers (HIA and MYH) for eligibility and this process was done individually, later, the discrepancy between them was resolved through discussion. The titles and abstracts of the studies were first checked during the search using eligibility criteria; later, the full text of all articles that might be included in this review was read. Table 2 contains articles that were excluded after reading the full text. Articles were excluded from this review when they did not meet one or more of the eligibility criteria.

**Table 2:** Articles that were excluded following full-text reading PAOO: periodontally accelerated osteogenic orthodontics

Study	Title of the paper	Reason for exclusion
Addanki et al., 2017 [23]	Clinical and Radiographic Comparative Evaluation of Buccal and Palatal Corticotomy with Buccal Corticotomy in Periodontally Accelerated Osteogenic Orthodontics with Surgical Bur	The therapeutic intervention was PAOO in both study groups
Thind et al., 2018 [24]	A clinical comparative evaluation of periodontally accelerated osteogenic orthodontics with piezo and surgical bur: An interdisciplinary approach	The therapeutic intervention was PAOO in both study groups
Liu et al., 2020 [25]	Membrane fixation for osseous graft stabilization in periodontally accelerated osteogenic orthodontics: a comparative study	The therapeutic intervention was PAOO in both study groups

Data collection process

Initially, a data extraction table for the included studies was formed. One reviewer (HIA) retrieved data from the included articles, and the other reviewer (MYH) examined those extracted data. And any discrepancy between them was resolved through dialogue and reference to the original article and back to the third reviewer (IA) to take the final decision. When there was a lack of data for the included studies, the authors were contacted by e-mail to obtain the required data. Several data were extracted from the studies included in the current review, which were the following: author's name, study design, year of publication, country of publication, sample size, gender, age of patients, type of malocclusion, and comprehensive treatment. Probing depth, bone density, root resorption, bone thickness, and marginal recession data were also obtained.

Risk of bias assessment

The two reviewers (HIA and MYH) independently assessed the risk of bias in all included studies using Cochrane's risk of bias tool for randomized trials (RoB2) and the ROBINS-I tool for non-randomized controlled trials [26,27]. Then, the judgments of both reviewers were compared, and a third reviewer (ASB) was asked to decide in case of disagreement. For randomized trials, the following fields were judged as having a high, low, or unclear risk of bias: Randomization process, deviations from intended interventions, missing outcome data, measurement of the outcome, and selection of the reported result. Then, the overall risk of bias for each trial was reported according to the following criteria: low risk of bias was reported if all fields were assessed as having a low risk of bias; moderate risk of bias was reported if one or more fields were assessed as having an unclear risk of bias; high risk of bias was reported if one or more fields were assessed as being at high risk of bias.

Summary measures, synthesis of results, additional analyses

The reviewers intended to pool data in a meta-analysis using Review Manager, Version 5.3 software. The mean, standard deviation, sample size, intervention, and outcomes were intended to be used to combine the results into a weighted mean difference (WMD) with 95% confidence intervals; the random-effects model was determined to be used for the continuous outcomes [22]. The P-value was planned to be used to detect any significant heterogeneity if P<0.05. The I^2^ index was intended to be employed to assess the percentage of heterogeneity [22].

Results

Study Selection

**Figure 1:**
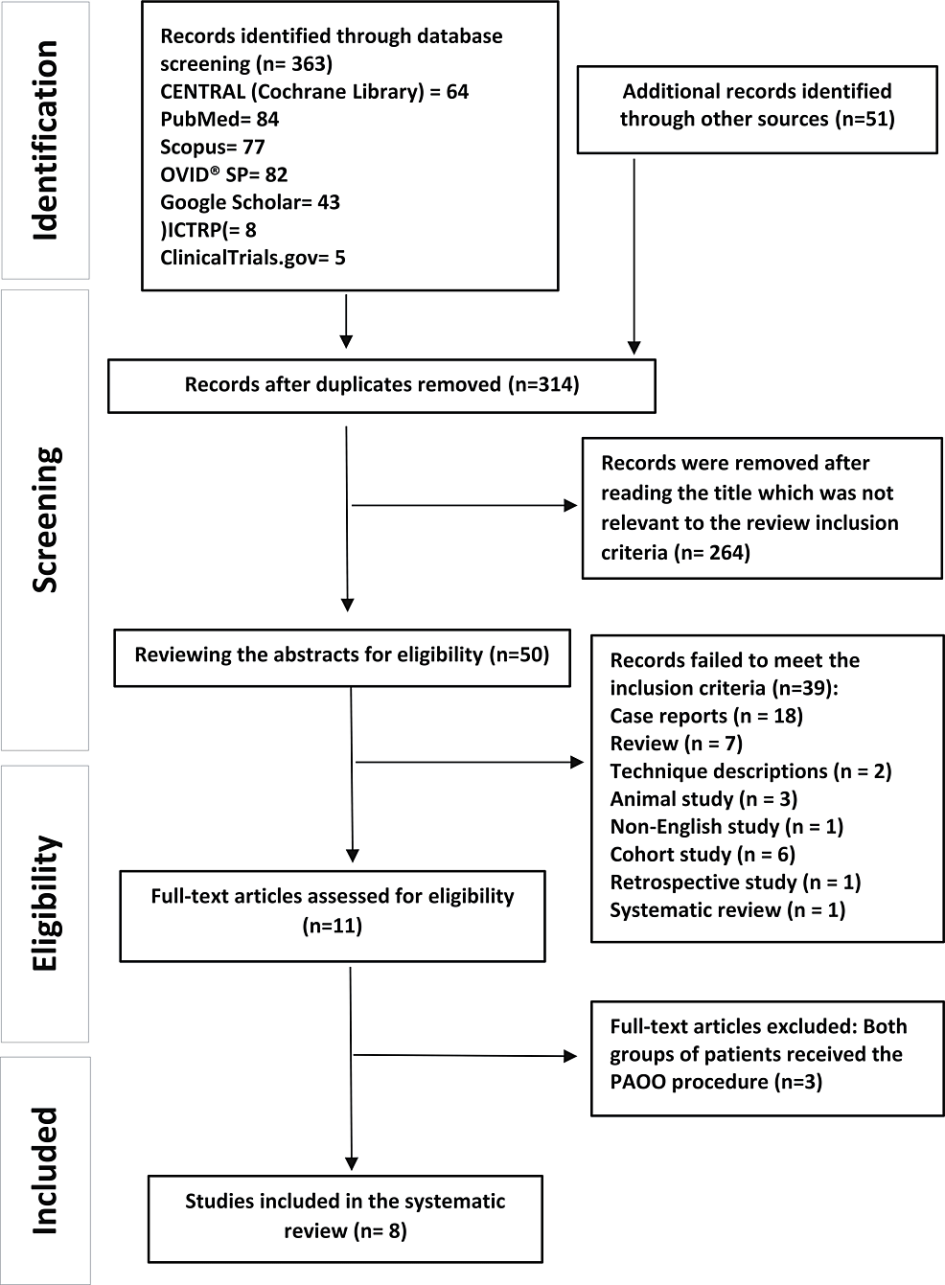
Preferred Reporting Items for Systematic Reviews and Meta-analyses (PRISMA) flow diagram of study identification, screening, eligibility and inclusion into the review. PAOO: periodontally accelerated osteogenic orthodontics

One thousand three hundred and ninety-three articles were found initially by all search queries. After excluding duplicates and articles by reading their titles, 50 papers remained for the second step. Thirty-nine articles were excluded after reading the abstract, and three articles were excluded after reading the full text. Finally, eight articles fulfilled the inclusion criteria and were included in the qualitative synthesis of the data. The PRISMA flow diagram is given in Figure 1.

Characteristics of the Retrieved Studies

Characteristics of the eight RCTs are given in Table 3. They are distributed under two sections: Section 1 included five RCTs that compared the PAOO procedure with conventional orthodontic treatment, i.e., without any adjunctive procedures [18,19,28-30], whereas Section 2 included three RCTs that compared the PAOO procedure with a corticotomy-assisted orthodontic treatment (CAOT) [17,31,32].

**Table 3:** Characteristics of included studies in this systematic review G: Group; PG: Parallel group; PAOO: Periodontally Accelerated Osteogenic Orthodontics; RCT: Randomized clinical trial; OT: Orthodontic Treatment; CO: Corticotomy; Tt: Treatment time; PD: Probing depth; BD: Bone density; RR: Root resorption; DFDBA: Demineralized Freeze-Dried Bone; BT: Bone thickness; DPD: Deoxypyridinoline; BMP-2: bone morphogenetic protein-2; VAS: Visual analog scale

Authors Country	Study design	Groups	Patients (M/F)	Mean age (SD)	Malocclusion	Extraction or without extraction	Bracket’s type or prescription	Bone graft	Radiographic evaluation
Shoreibah et al., 2012; Egypt [32]	RCT (2-arm PG)	GI: OT+CO GII: OT+PAOO	(M/F): 20 (4/16) GI:10 GII: 10	GI: 24.6 years GII: 24.6 years	Class I with moderate lower anterior crowding 3-5mm	GI/GII: Without extraction	Roth prescription 0.022*0.028-inch slot	GII: Bioactive glass	Panoramic radiographs + Lateral Cephalogram
Abbas and Moutamed, 2012; Egypt [28]	RCT (2-arm PG)	GI: OT GII: OT+PAOO	(F): 8 GI: 4 GII: 4	GI: 22.3 (2.26) years GII: 22.3 (2.26) years	Class I with moderate lower anterior crowding	GI/GII: Without extraction	Not reported	GII: Bioglass granules	Lateral Cephalogram + Periapical radiographs
Bhattacharya et al., 2014; India [30]	RCT (2-arm PG)	GI: OT GII: OT+PAOO	(M/F): 20 (2/18) GI: 10 GII: 10	GI: 19,8 (3.22) years GII: 18,8 (3,48) years	Any case of malocclusion requiring retraction of upper anterior teeth	GI/GII: Extraction of the upper 1^st^ premolars	MBT prescription 0.022-inch slot	GII: DFDBA	Panoramic radiographs + Lateral Cephalogram + CT scan for maxillary only
Al-Naoum et al., 2015; Syria [19]	RCT (2-arm PG)	GI: OT GII: OT+PAOO	(M/F): 30 (13/17) GI: 15 GII: 15	GI: 20.43 (2.67) years GII: 20.43 (2.67) years	Class I with severe anterior crowding 5-7mm	GI: Extraction of the upper/lower 1^st^ premolars, GII: Without Extraction	Roth prescription 0.022*0.028-inch slot	GII: Bovine bone grafts	Panoramic radiographs + Lateral Cephalogram
Wu et al., 2015; China [18]	A pilot clinical study (2-arm PG)	GI: OT GII: OT+PAOO	(M/F): 24 (8/16) GI: 12 GII: 12	GI: 20.6 (2.0) years GII: 20.1 (1.6) years	Skeletal Class III surgical patients with mild upper anterior crowding	GI/GII: Extraction of the upper 1^st^ premolars	Edgewise prescription 0.022-inch slot	GII: Tricalcium phosphate	Lateral Cephalogram
Aristizabal et al., 2016; Colombia [29]	A pilot clinical study (2-arm PG)	GI: OT GII: OT+PAOO	(M): 10 GI: 5 GII: 5	GI: 28.5 (6.3) years GII: 29.6 (9.8) years	Class I/II, with mild anterior crowding	GI/GII: Without Extraction	Damon Q self-ligating braces	GII: Allograft	Panoramic radiographs + Lateral Cephalogram + CBCT
Bahammam, 2016; Saudi Arabia [17]	RCT (3-arm PG)	GI: OT+CO GII: OT+PAOO GIII: OT+PAOO	(M/F): 33 (13/20) GI:11 GII:11 GIII:11	GI: 21.2 (1.43) years GII: 21.2 (1.43) years GIII: 21.2 (1.43) years	Class I with moderate lower anterior crowding	GI/GII/GIII: Without Extraction	Roth prescription 0.022*0.028-inch slot	GII: Bovine bone grafts GIII: Bioactive glass	Lateral Cephalogram + Periapical radiographs
Chandra et al., 2019; India [31]	RCT (2-arm PG)	GI: OT+CO GII: OT+PAOO	M/F): 30 (13/17) GI:15 GII:15	GI: 23.62 (6.23) years GII: 23.62 (6.23) years	Class I with moderate lower anterior crowding	GI/GII: Extraction of the lower 1^st^ premolars	Not reported	GII: BMP-2	Lateral cephalogram + Periapical radiographs

The eight studies included 175 participants (63 males, 112 females), 93 patients treated with PAOO, 36 patients with CAOT, and 46 patients with orthodontic treatment without any adjuvant procedure. The average age ranged from 18.8 to 29.6 years. One study included only female patients [28]. Another study included only male patients [29], whereas the remaining six studies included females and males, with a predominance of female patients [17-19,30-32]. Two clinical trials were from Egypt [28,32] and two articles from India [30,31], while one article from each of the following countries: Syria [19], China [18], Colombia [29], and Saudi Arabia [17]. Seven studies were on two-arm parallel-group designs [18,19,28-32], whereas one study was a three-arm parallel-group trial [17].

Six trials evaluated the decrowding of lower and/or upper anterior teeth [17,19,29,31-33]. Four of them included non-extraction treatment with minimal to moderate maxillary and/or mandibular anterior crowding [17,28,29,32]. In contrast, the remaining two trials involved either extracting the lower first premolars [31] or extracting the upper and lower first premolars [19]. The last two clinical trials involved retraction of the upper anterior teeth after extraction of the first upper premolars [18,30]. Two trials included the traditional surgical procedure described by Wilcko et al. [14], by elevating a full-thickness flap on the buccal and palatal side [28,30]. However, six articles included an adjustment to the surgical procedure by elevating the buccal flap only [17-19,29,31,32]. Selective cortical cutting in the PAOO was performed using a stainless steel surgical bur in four trials [17,30-32]. Nevertheless, a piezosurgery unit was used in the other four trials [18,19,28,29].

Regarding bone grafts, a variety of bone grafts were used. Allograft was employed in two trials [29,30], xenograft in two trials [17,19], synthetic bone substitutes in four trials [17,18,28,32] and composite grafts in one trial [31]. Regarding the primary outcomes, eight articles assessed the duration of orthodontic treatment [17-19,28-32], three articles assessed bone density using periapical radiographs [17,31] or panoramic radiographs [32], and one article assessed the alveolar thickness using a CT scan [27]. On the other hand, the secondary outcomes included clinical probing depth assessment in three studies and were dependent on using a periodontal probe [17,29,32]. Evaluation of root resorption was undertaken in two papers with the aid of panoramic radiographs [32] or periapical radiographs [17]. Only one study evaluated pain levels postoperatively using a VAS tool [31].

Risk of Bias in the Included Studies

**Figure 2:**
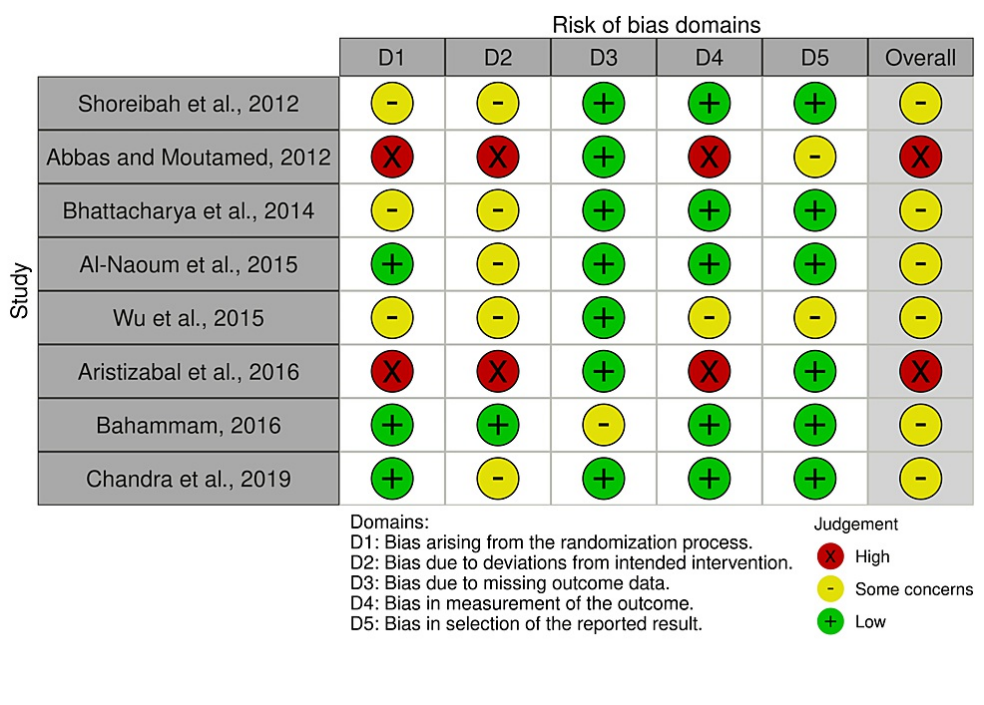
Assessment of the risk of bias in each study included in this review. The risk of bias figure contains eight studies with the following authors: Shoreibah et al. [32], Abbas and Moutamed [28], Bhattacharya et al. [30], Al-Naoum et al. [19], Wu et al. [18], Aristizabal et al. [29], Bahammam [17], and Chandra et al. [31].

**Figure 3:**
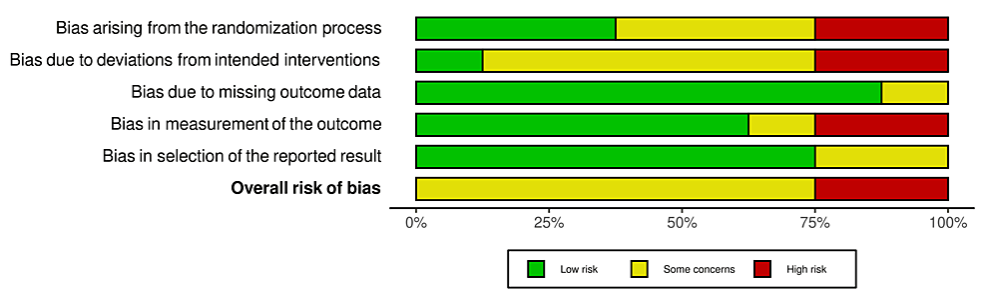
The overall risk of bias in the included studies.

The summary of the risk of bias of the included RCTs is shown in Figure 2 and Figure 3. Three RCTs were at unclear risk of bias, three RCTs were at low risk of bias, and two RCTs were at high risk of bias. More problems were notedFigureFigure about the concealment of allocation; the articles with a high risk of bias were 25%, and the articles with some concerns were 37.5%. The participants' blinding was another problem seen as a high risk of bias in 25% of the included studies and as having some concerns in 62.5% of the included studies. More information about the risk of bias assessment, along with the reasons supporting each assessment, can be found in Table 4.

**Table 4:** Risk of bias of the included randomized controlled trials with explanations for each judge.

Study	D1	D2	D3	D4	D5	Overall bais
Shoreibah et al., 2012 [32]	Some concerns: The patients were randomly divided into two groups, without mentioning the method of randomization	Some concerns: Blinding of participants and people delivering the intervention cannot be performed.	Low risk: No patient was lost to follow-up.	Low risk: No details of blinding of outcome assessors. But we judge that the outcome was not likely to be influenced by knowledge of intervention received	Low risk: The protocol was not registered. But the pre-defined outcomes mentioned in the methods section seemed to have been reported	Some concerns: The study is judged to raise some concerns because two domains get this result
Abbas and Moutamed, 2012 [28]	High risk: The patients were randomly divided into two groups, but we judged that randomization was biased	High risk: Blinding of either patient or clinician was not possible.	Low risk: No dropouts were reported	High risk: No details of blinding of outcome assessors. But we judge that the outcome was likely to be influenced by knowledge of intervention received	Some concerns: The protocol was not registered. However, not all outcome variables to be studied may not have been reported	High risk: The study was judged to be high risk because three domains get this result
Bhattacharya et al., 2014 [30]	Some concerns: The patients were randomly divided into two groups, without mentioning the method of randomization	Some concerns: Blinding of participants and people delivering the intervention cannot be performed.	Low risk: No dropouts were reported	Low risk: No details of blinding of outcome assessors. But we judge that the outcome was not likely to be influenced by knowledge of intervention received	Low risk: The protocol was not registered. But the pre-defined outcomes mentioned in the methods section seemed to have been reported	Some concerns: The study is judged to raise some concerns because two domains get this result
Al-Naoum et al., 2015 [19]	Low risk: Randomization sequences were generated using sealed envelopes containing the random allocation of each patient to one or the other group.	Some concerns: Blinding of participants and people delivering the intervention cannot be performed.	Low risk: No dropouts were reported	Low risk: No details of blinding of outcome assessors. But we judge that the outcome was not likely to be influenced by knowledge of intervention received	Low risk: The protocol was not registered. But the pre-defined outcomes mentioned in the methods section seemed to have been reported	Some concerns: The study is judged to raise some concerns because one domain gets this result
Wu et al., 2015 [18]	High risk: Patients were not randomized	High risk: Blinding of either patient or clinician was not possible.	Low risk: No dropouts were reported	Low risk: No details of blinding of outcome assessors. But we judge that the outcome was not likely to be influenced by knowledge of intervention received	Low risk: The protocol was not registered, it is a preliminary study that does not necessarily need to be registered	High risk: The study was judged to be high risk because two domains get this result
Aristizabal et al., 2016 [29]	High risk: Patients were not randomized	High risk: Blinding of either patient or clinician was not possible.	Low risk: No patient was lost to follow-up.	High risk: No details of blinding of outcome assessors. But we judge that the outcome was likely to be influenced by knowledge of intervention received	Low risk: The protocol was not registered, it is a preliminary study that does not necessarily need to be registered	High risk: The study was judged to be high risk because three domains get this result
Bahammam, 2016 [17]	Low risk: Patients were randomly divided into two groups, and the study was judged to be of low risk	Low risk: Blinding of participants and people delivering the intervention cannot be performed. But the study was judged to be of low risk	Some concerns: Some patients were lost during the follow-up period, and the study was judged to be of Some concerns	Low risk: No details of blinding of outcome assessors. But we judge that the outcome was not likely to be influenced by knowledge of intervention received	Low risk: The protocol for the study was registered in clinical trial.gov study ID: (NCT02796911) and the outcomes that mentioned in the protocol have been reported	Some concerns: The study is judged to raise some concerns because one domain gets this result
Chandra et al., 2019 [31]	Low risk: Patients were randomly divided into two groups, and the study was judged to be of low risk	Some concerns: Blinding of participants and people delivering the intervention cannot be performed.	Low risk: No dropouts were reported	Low risk: No details of blinding of outcome assessors. But we judge that the outcome was not likely to be influenced by knowledge of intervention received	Low risk: The protocol for the study was registered in clinical trial.gov study ID: (NCT03396900) and the outcomes that mentioned in the protocol have been reported	Some concerns: The study is judged to raise some concerns because one domain gets this result

Orthodontic Treatment Effects of the PAOO Procedure Versus no Adjunctive Procedure

Treatment time: Three clinical studies indicated that PAOO caused an acceleration of leveling and alignment (39%, 246 versus 402 days; 46%, 171.9 versus 314 days; 47%, 74.5 versus 141.7 days; respectively) when compared with conventional orthodontic treatment [19,28,29]. Two studies indicated that the PAOO caused an acceleration of retraction of the upper anterior teeth (44%, 156 versus 441 days; and 61%, 130.5 versus 234.1 days; respectively) when compared with conventional orthodontic treatment [18,30], as shown in Table 5.

**Table 5:** The main findings of the included studies in this systematic review G: Group; PG: Parallel group; PAOO: Periodontally Accelerated Osteogenic Orthodontics; RCT: Randomized clinical trial; OT: Orthodontic Treatment; CO: Corticotomy; Tt: Treatment time; PD: Probing depth; BD: Bone density; RR: Root resorption; DFDBA: Demineralized Freeze-Dried Bone; BT: Bone thickness; DPD: Deoxypyridinoline; BMP-2: bone morphogenetic protein-2; VAS: Visual analog scale

Authors, Study design, and C ountry	Groups	Outcomes	Surgical intervention	Orthodontic treatment starts/adjustment periods	Results	Conclusion	Follow-up
Shoreibah et al., 2012; RCT; Egypt [32]	GI: OT+CO GII: OT+PAOO	Treatment time Probing depth Bone density Root resorption	Full-thickness flaps were reflected labially from the distal surface of the lower right canine to the distal surface of the lower left canine, vertical decortication was performed using a small round stainless steel surgical bur	Immediately after the surgical procedure / every 2 weeks	The treatment time in the GII was 17 weeks vs 16.67 weeks in the GI. Regarding probing depth and root resorption, there was no statistical difference between the two treatment groups. During the period between preoperative and six months post-treatment, bone density decreased in group I by 17.59% vs an increase of 25.85% in group II.	PAOO reduced the total treatment time, increased the alveolar bone density, and reduced the incidence of root resorption and periodontal problems	6 months after debonding
Abbas and Moutamed, 2012; RCT; Egypt [28]	GI: OT GII: OT+PAOO	Treatment time	A full-thickness flap was reflected on both buccal and lingual sides, extending at least two to three teeth beyond the area to be treated. Vertical decortication was performed in the inter-radicular spaces and horizontal decortication was also accomplished using a piezo ultrasonic surgery unit	After 2 weeks of surgery / every 2 weeks	Decrowding was achieved in 74.5±7.7 days in GII vs 141.7±21.3 days in GI. The average duration of mandibular decrowding treatment in GII was reduced by about 50%.	PAOO reduced the treatment time	until debonding
Bhattacharya et al., 2014; RCT; India [30]	GI: OT GII: OT+PAOO	Treatment time Alveolar bone thickness	A full-thickness flap was reflected on both buccal and lingual sides, from the mesial area of the right 1^st^ premolar to the mesial area of the left 1^st^ premolar. Vertical decortication was performed in the inter-radicular spaces and horizontal decortication was also accomplished, using a round surgical bur	After 2 weeks of surgery / not reported	The retraction time in the GII was 130.5 days vs 234.1 days in the GI. There was a significant difference in total alveolar bone thickness at the crest region for all four incisor teeth (p<0.05). A significant difference was observed in total alveolar bone thickness at the S2 and S3 level for 11, 21 and 11, 12 and 22 (p<0.05) respectively.	Alveolar corticotomy not only accelerates the orthodontic treatment but, also provides the advantage of increased alveolar width to support the teeth and overlying structures	until debonding
Al-Naoum et al., 2015; RCT; Syria [19]	GI: OT GII: OT+PAOO	Treatment time, E- line, Little Index, inter-canines-width, inter-2^nd^ premolars-width, incisors inclination, arch length	Full-thickness flaps were reflected labially from the distal surface of the right second premolar to the distal area of the left second premolar. Vertical decortication and small round corticotomy perforations were performed using a piezo ultrasonic surgery unit	After 2 weeks of surgery / every 2 weeks	The alignment and leveling stages took 5.73 months in the PAOO group while they took 10.48 months in the extraction group	PAOO procedure provides a safe alternative for patients with moderate to severe crowding who desire the benefits of orthodontic treatment in a relatively short period.	until debonding
Wu et al., 2015; RCT; China [18]	GI: OT GII: OT+PAOO	Time duration Measurement of virtual 3D maxillary model Measurement inter-upper canines-width, measurement inter-1^st^ upper molars-width	A full-thickness flap was reflected labially from the mesial area of the right second premolar to the mesial area of the left second premolar. Vertical decortication was performed using a piezo ultrasonic surgery unit	After 2 weeks of surgery / every 4 weeks	PAOO significantly shortened the extraction space closure period P<0.001. No significant statistical difference between the two treatment groups concerning all measurements of the 3D virtual maxillary models	PAOO can reduce the preoperative orthodontic treatment time in leveling, alignment, and extraction space closure phase for the skeletal Class III surgical patients by an average of more than half a year. The PAOO procedures do not save anchorage	until debonding
Aristizabal et al., 2016; RCT; Colombia [29]	GI: OT GII: OT+PAOO	Duration treatment Probing depth Marginal recession Deoxypyridinoline	Full-thickness flaps were reflected labially on the upper and lower jaws, vertical decortication was performed using a piezo ultrasonic surgery unit	After 2 weeks of surgery / every 2 weeks	The total treatment time for the experimental group was 8.2±3.3 months and for the control group was 13.4±7.3 months. The type of treatment showed no differences in periodontal initial and final conditions	There is a reduction in treatment time for patients undergoing PAOO with DAMON Q braces. There is no difference in the periodontal condition between PAOO and conventional orthodontics	6 months after debonding
Bahammam, 2016; RCT; Saudi Arabia [17]	GI: OT+CO GII: OT+PAOO GIII: OT+PAOO	Duration treatment Probing depth Bone density Root resorption	A full-thickness flap was reflected labially from the distal surface of the lower right canine to the distal surface of the lower left canine. Selective alveolar decortication was performed through the labial cortical plate of bone, using a small round stainless steel surgical bur.	After 2 weeks of surgery / every 2 weeks	Orthodontic treatment was reduced to an average of 11.4 ± 0.14 weeks in all groups. There was no evidence of significant apical root resorption at any time interval. All groups showed a decrease in mean bone density at debonding followed by an increase at 9 months post-treatment. GII and GIII showed a statistically significant increase in a bone density greater than GI at 9 months post-treatment	PAOO decreases the duration of active treatment and reduces the risk of root resorption	9 months after debonding
Chandra et al., 2019; RCT; India [31]	GI: OT+CO GII: OT+PAOO	Duration treatment Bone density Visual Analog Scale (VAS)	A full-thickness flap was reflected labially from the mesial surface of the lower right second premolar to the mesial surface of the lower left second premolar. Selective alveolar decortication was performed through the labial cortical plate of bone, using A701 surgical bur.	Immediately after the surgical procedure / every 2 weeks	There was no significant difference in the mean Little’s index values between both the groups. After 6 months of starting orthodontic treatment, there was a statistically significant difference in bone density (P≤0.05) between both the treatment groups	BMP‑2 has the potential to function as a regenerative material in PAOO	until debonding

Alveolar bone thickness and periodontal changes following treatment: One trial only evaluated the effectiveness of PAOO in increasing the thickness of the alveolar bone [30]. Bhattacharya et al. reported that the alveolar bone thickness increased by 0.11 to 0.46 mm at the end of the extraction space closure stage in the PAOO group, at the level of the maxillary anterior incisors, and the increase in alveolar bone thickness was statistically significant at the cervical, the middle and the apical third of the root (P<0.001, P<0.001, P=0.004 respectively). Only one trial evaluated the effect of PAOO on periodontal status [29] and found that changes in probing depth and marginal recession in the PAOO group were not significantly different compared to the control group.

Orthodontic Treatment Effects of the PAOO Versus Corticotomy-Only as an Adjunctive Procedure

Treatment time:** **Two trials evaluated the effectiveness of the PAOO procedure in reducing treatment time when leveling and aligning moderately crowded lower anterior teeth compared to a corticotomy-only group [31,32]. Shoreibah et al. found no significant difference between the two groups (117 versus 119 days, P>0.05), but Chandra et al. reported a significant difference between the two groups (71.7 versus 102.9 days, P<0.001). One three-arm clinical trial assessed the effectiveness of the PAOO in reducing treatment time when leveling and aligning moderately crowded lower anterior teeth. The study included two PAOO groups (one with xenograft and one with bioactive glass) that were compared to the corticotomy-only group and found no significant differences among the three groups in this variable (117.6 versus 100.8 versus 105 days, respectively; P>0.05) [17].

Bone density: Two clinical trials reported that the bone density decreased at the end of treatment in the PAOO group as well as in the corticotomy-only group [17,32], but that bone density increased at the end of the follow-up period in the PAOO group significantly versus the corticotomy-only group (at nine months: P<0.001; at six months: P=0.001).

One clinical trial reported that bone density increased in the PAOO group and the corticotomy-only group at three and six months following the onset of orthodontic treatment [31]. However, the increase in the PAOO group with significantly greater than in the corticotomy-only group at six months of treatment (626.56±122.72, 429.4±118.46, P=0.013, respectively).

Periodontal changes following treatment: Two trials evaluated the effect of PAOO on periodontal status [17,32]. Bahammam found that the probing depth was decreased in the corticotomy-only group more than the value of the probing depth in the PAOO group with xenograft, as well as compared with the group PAOO with bioactive glass at the end and after nine months of treatment, with a statistically significant difference between the three treatment groups (P<0.05). Shoreibh et al. showed that the probing depth did not differ statistically between the two treatment groups.

Root resorption: Two articles evaluated the effect of PAOO on root resorption [17,32]. The former study used periapical digital radiography [17], whereas the latter used panoramic radiographs [32]. Both clinical trials indicated no statistically significant difference between the PAOO group and the corticotomy-only group in root resorption at the end of the orthodontic treatment and follow-up period.

Perception of pain: Only one article evaluated the level of pain assessed on the VAS scale [31]. According to Chandra et al., there was no statistical difference in the level of pain in the immediate postoperative period and at the end of the second week in the PAOO group compared to the corticotomy group. However, at the end of the first week, there was a decrease in the level of pain in the PAOO group statistically compared to the corticotomy group.

Discussion

This is the first systematic review of the effectiveness of PAOO in comparison with two types of intervention, traditional orthodontic and corticotomy-only, as an adjunctive procedure. The current review conducted a comprehensive evaluation of the literature published in most of the available databases on the efficacy of PAOO. This review included 175 patients from eight clinical trials. Randomized controlled clinical studies were included to reduce potential bias and confounding. All the included clinical trials evaluated the effectiveness of PAOO in accelerating tooth movement, in addition to five outcome variables (alveolar bone thickness, periodontal changes, bone density, root resorption, pain, and discomfort levels).

Orthodontic Treatment With the PAOO Versus no Adjunctive Procedure

Treatment time: Three clinical trials demonstrated the efficacy of PAOO in accelerating leveling and alignment [19,28,29], and two studies indicated its efficacy in accelerating the retraction of the upper anterior teeth compared to conventional orthodontic treatment [18,30]. This acceleration was explained by the surgical intervention that induced the RAP, which begins within days of the surgical injury and reaches its peak within one to two months of the surgical intervention, and takes six to 24 months to end [14]. In the study of Alfawal et al., the RAP peaked one month after the surgical intervention and then decreased gradually at the end of the second month [10]. The conservative surgical intervention explained the peak of acceleration at one month and the regression in the following month.

One of the previously mentioned studies reported no statistically significant difference in acceleration between the PAOO procedure and the traditional orthodontic treatment [29]. The absence of a statistical difference between the two groups may be explained by the high bias in the patient selection, the choice of the treatment intervention, and the use of an additional accelerator method, which was self-ligating orthodontic brackets.

Alveolar bone thickness and periodontal changes following treatment: One clinical trial indicated the effectiveness of PAOO in increasing bone thickness at the level of the maxillary anterior incisors within the same group, i.e., before and after treatment [30]. Wilcko et al. also claimed the efficacy of PAOO in increasing the width and thickness of the alveolar bone and thus healing bony defects [14]. Therefore, in the future, more clinical trials are needed to investigate this outcome.

Only one trial evaluated the effect of PAOO on periodontal status and found that changes in probing depth and marginal recession in the PAOO group were not significantly different compared to the control group [29]. In the study of Ma et al., they indicated that probing depth did not change significantly, but about gingival recession, it improved significantly (P<0.001) within the PAOO group before and after three and 12 months of follow-up [31]. Therefore, in the future, more clinical trials are needed to investigate these outcomes.

The PAOO Versus Corticotomy-Only as an Adjunctive Procedure

Treatment time:** **The three trials evaluated the effectiveness of PAOO procedures in reducing the duration of orthodontic treatment versus corticotomy-only [17,31,32]. The difference was not significant in the two clinical trials [17,32]. But Chandra et al., in their clinical trial, indicated that PAOO with bone morphogenetic protein-2 (BMP‑2) significantly reduced treatment time versus corticotomy-only [31]. They explained that the decrease in mineralization occurs to a greater extent when BMP-2 is applied, which reduces the resistance of the alveolar bone to dental movement and leads to a decrease in the duration of orthodontic treatment [34].

Bone density: Two clinical trials reported that the bone density decreased at the end of treatment in the PAOO group as well as in the corticotomy-only group [17,32], which is explained by surgical trauma that induces localized osteoporosis [17]. However, the two previous studies indicated that the alveolar bone density increased significantly at the end of the follow-up period [17,32]. This may be explained by the incorporation of the bone graft with the cortical bone [29], and other studies have indicated that incorporation of the bone graft into the new bone layer has a beneficial effect in repairing the bone defect and increasing the volume of the alveolar bone [14,35]. Whereas one clinical trial [31] reported that bone density increased in the PAOO group as well as in the corticotomy-only group at three and six months following the onset of orthodontic treatment, which was explained by the high osteoinductive property of BMP‑2 [31].

Periodontal changes following treatment:** **Two trials evaluated the effect of PAOO on periodontal status [17,32]. The changes in probing depth in the groups subjected to the PAOO procedures were not significantly different compared to the corticotomy-only groups. Also, the patients showed a slight improvement in the value of probing depth and this is consistent with previous studies [14,36]. No study evaluated gingival index, plaque index, and bleeding on probing, dental pulp test, clinical attachment level, or the width of attached gingiva. Therefore, further studies are needed to assess periodontal status after surgical intervention associated with undergoing PAOO.

Root resorption: Two articles evaluated the effect of PAOO on root resorption [17,32]. Both clinical trials indicated that there was no statistically significant difference between the PAOO group and the corticotomy-only group in root resorption. This was explained by the shortening of the period of orthodontic treatment as a result of corticotomy in both groups [14,32,36,37] because corticotomy procedures reduce bone density and thus accelerate tooth movement [32].

Perception of pain: Only one article evaluated the level of pain assessed on a VAS scale [31]. This assessment was made in the immediate postoperative period. However, this may not have reflected the actual level of pain due to the possible remaining effect of the anesthetic used at the time of assessment. In addition, their study missed the evaluation of pain levels at one, two, three, or even five days following surgery. Therefore, the information provided by this study was not clinically useful. This increases the need for studies evaluating levels of pain, discomfort, and functional impairments over multiple postoperative assessment points, as well as patient satisfaction with PAOO therapy.

Limitation of the Current Review

The current review targets articles written in English only, which can be considered a limitation of this review. A small number of studies was retrieved in this systematic review, and the reported results were not consistent among studies. Therefore, there is a need for more high-quality randomized controlled clinical trials on the efficacy of the PAOO procedure in accelerating tooth movement. The high heterogeneity among the included studies as well as the use of different outcome measures and treatment protocols, prevented the conduction of a meta-analysis. Side effects and significant complications were not clearly covered in the evaluated papers despite the apparently aggressive nature of the PAOO procedure. Future research work should focus on the untoward effects that accompany surgical interventions.

## Conclusions

Based on the evidence derived from the retrieved papers, the PAOO procedure was effective in accelerating orthodontic tooth movement. The evidence came from several studies with different levels of the risk of bias indicating the need for more high-quality randomized controlled clinical trials on the effectiveness of the PAOO procedure in comparison with non-accelerated traditional orthodontic treatment or with accelerated treatments employing other methods. The PAOO procedure tended to increase the thickness of the alveolar bone although the evidence in this regard requires additional studies. Most of the periodontal outcome measures in relation to PAOO application were not thoroughly covered in the included trials. Levels of pain, discomfort, and acceptance associated with surgical intervention were not systematically studied over the observation period.
